# A Modified Ascending Aortic Cannulation Technique in Minimally Invasive Totally Thoracoscopic Cardiac Surgery

**DOI:** 10.21470/1678-9741-2021-0234

**Published:** 2022

**Authors:** Shengjie Liao, Xiaoshen Zhang

**Affiliations:** 1 Department of Cardiovascular Surgery, the First Affiliated Hospital of Jinan University, Guangzhou, China.

**Keywords:** Cardiopulmonary Bypass, Aortic Aneurysm, Minimally Invasive Surgery Procedures, Femoral Artery

## Abstract

Cannulation through the femoral artery is the preferred method of establishing peripheral cardiopulmonary bypass in minimally invasive totally thoracoscopic cardiac surgery. However, faced with the contraindication of femoral artery cannulation, modified ascending aortic cannulation is an alternative approach to minimally invasive totally thoracoscopic cardiac surgery.

**Table:** Abbreviations, Acronyms & Symbols

CT	= Computed tomography

## INTRODUCTION

Minimally invasive totally thoracoscopic cardiac surgery has the advantages of less trauma, quick recovery and early postoperative activities, so it has been widely accepted worldwide^[1]^. The establishment of peripheral cardiopulmonary bypass is essential. At present, femoral artery cannulation is the most frequently used approach. However, more alternative choices should be provided as there were contraindications including vascular variation, calcification, plaque, and mural thrombus. This article aims to present a modified traditional aortic cannulation approach adopted in minimally invasive totally thoracoscopic surgery. No informed consent statement was required for this technique.

### Case Report

A 60-year-old male with severe mitral regurgitation, severe tricuspid regurgitation, and atrial fibrillation, was planned to undergo mitral valve repair and tricuspid valve repair under minimally invasive totally thoracoscopic surgery. Informed consent was obtained from the patient during the perioperative dialogue. Preoperative computed tomography (CT) scan of the aorta showed that there were some mural thrombi in the abdominal aorta and bilateral iliac arteries, which contraindicated femoral artery cannulation since the risk of thrombus detachment and embolism was high. Therefore, ascending aortic cannulation was chosen according to his cardiovascular conditions.

After general anesthesia and double-lumen endotracheal intubation, the patient was adjusted to a partial 30-degree right lateral position with the right arm suspended above the head to fully expose the intercostal spaces. After systemic heparinization, a 28Fr femoral vein cannula was placed into the right atrium through the femoral vein. There was a main port in the right 4^th^ intercostal space, a camera port in the 5^th^ intercostal space, an assist port in the 3^rd^ intercostal space, and another 5 mm incision for the ascending aortic cannula through the 2^nd^ intercostal space when the central cannulation was completed.

**Fig. 1:**
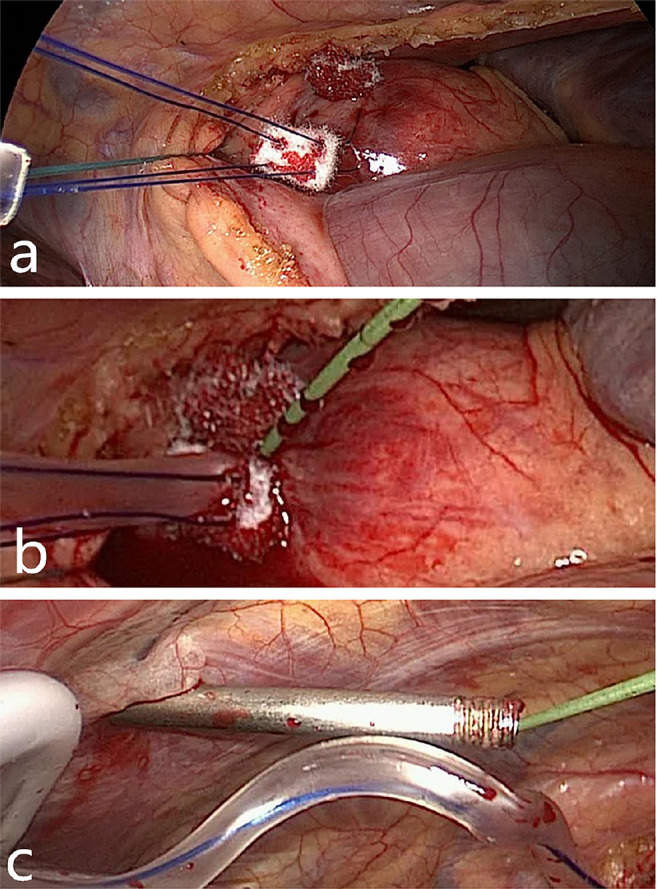
(a) Purse-string suture. (b) Needle punctured into the aorta to insert the guidewire. (c) The introducer leads the guidewire out of the body.

**Fig. 2:**
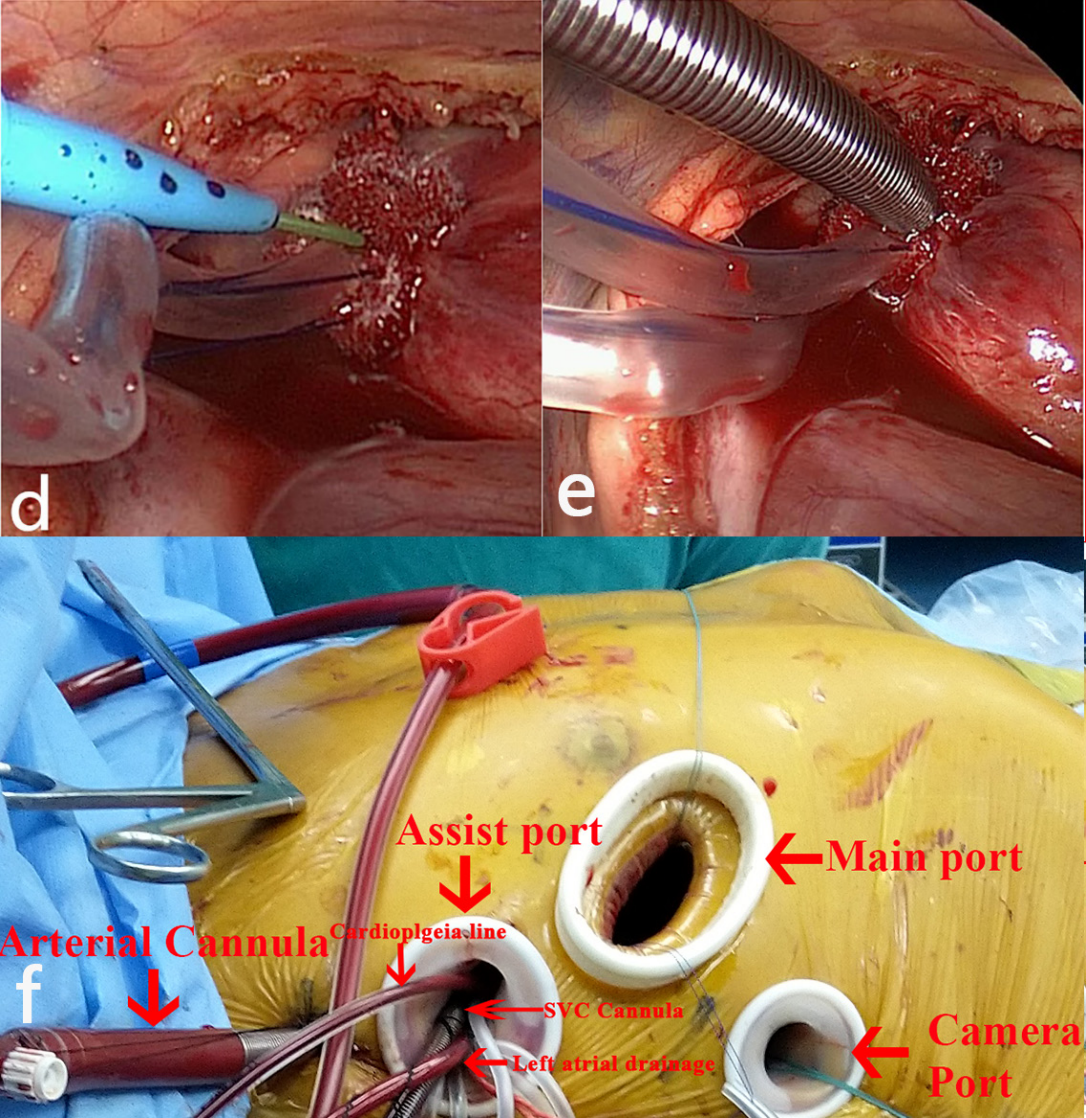
(d) Cannula introduced along the guidewire. (e) Aortic cannula adjusted and secured. (f) Schematic diagram of the surgical incision and cannula location.

Two 3-0 polypropylene purse-string sutures with pledgets were placed in the ascending aorta with the free ends controlled with tourniquets on each side. A puncture needle was inserted into the ascending aorta through the main port and the guidewire was pushed through the needle into the aorta. An introducer was inserted into the thoracic cavity through the right 2^nd^ intercostal space of the anterior axillary line to lead the tail of the guidewire out of the body, as shown in Figure 1. The cannula (16Fr femoral artery cannula) was gently inserted into the aorta along the guidewire with the tip toward the aortic arch to avoid injury to the posterior wall of the aorta. The cannula was carefully fixed, as shown in Figure 2. After finishing the surgery, the aortic cannula was pulled out and the purse-string suture was tightened to control the bleeding.

## DISCUSSION

With the aging of the population and changes in lifestyle and dietary habits, the incidence of calcification in the cannulation position is increasing^[2]^. Meanwhile, arterial mural thrombi formation may occur in normal vessels and atherosclerotic arteries, even in the absence of a hypercoagulative state, inflammation, or infection^[3,4]^. In the presence of severe atherosclerosis, stenosis, distorted deformities, or arterial mural thrombi, cannulation into the target or adjacent artery is contraindicated. Therefore, vascular ultrasonography or CT scan of the aorta should be completed before surgery to determine whether there is a contraindication to peripheral cannulation.

Cannulation of the femoral artery is preferred in minimally invasive cardiac surgery, which has the advantages of convenience, quickness, low complication rate and ease of operation^[4]^. Given the contraindication of femoral artery cannulation, another approach should be considered. Subclavian artery cannulation is another good option that has the advantage of providing forward blood flow without affecting the view of the surgical field. However, an extra incision is required. Moreover, the subclavian artery is not easily accessible due to its deep location, complex surrounding anatomy, and higher complication rate.

Cannulation of the ascending aorta is a conventional procedure for approaching a median sternotomy, mini-sternotomy or right anterior thoracotomy. It can be done easily under fully exposure and short surgical distance. But it would be a disaster if it were applied to minimally invasive cardiac surgery without any modification, especially in minimally invasive totally thoracoscopic surgery or robotic minimally invasive cardiac surgery. With the limited skin incision and soft tissue retractor alone, there would be no possibility of directly seeing or touching the aorta. With the long distance and limited surgical view and space in minimally invasive totally thoracoscopic surgery, a higher rate of complications like massive hematoma, aortic dissection, and cannulation failure caused by the failure to control aortic hemorrhage in time may happen if conventional technique is applied. Thus, a modified aortic cannulation method guided by a previous inserted guidewire was adopted. The needle puncture point will not have much blood coming out, and with the help of the guidewire, the central aorta cannulation will be more reliable and easier.

The Seldinger technique was also described in the review by Lamelas^[5]^, in which suitable patients with the aorta shifted to the right, a larger skin incision and rib retractor had to undergo this modified technique. Moreover, it may hinder the already limited surgical field if the cannula coming through the main incision. The modified technique described in this article is relatively simple and the probability of complication is largely decreased. With the purse-strings secured with the tourniquets during the removal of the arterial cannula, an acute hemorrhagic complication may not occur under normal circumstances. But if this occur, the insertion point must be blocked by a peanut-like gauze or partial clamping to stop the acute bleeding. After that, a purse-string with multiple pledgets is required to repair the damage by a surgeon with extensive expertise in minimally invasive cardiac surgery. If this does not work, a median sternotomy is strongly recommended.

## CONCLUSION

This modified technique can be accomplished by an experienced surgeon with just 5 mm of extra incision, no hinder to exposure, no need for specific aortic guidance, and lower complication rate of central aortic cannulation. The technique would be a potential solution for patients facing contraindication for femoral artery cannulation, especially in minimally invasive totally thoracoscopic surgery or robotic minimally invasive cardiac surgery. However, it does require the surgeon to have extensive experience and skill in thoracoscopy. This modified technique is not recommended to the beginner surgeons or surgeons with less experience in minimally invasive cardiac surgery. This is the last choice for cannulating the ascending aorta in minimally invasive cardiac surgery. Remaining clinical effects and complications still need to be observed in long-term, large-scale cases.
